# Effects of Aerobic Exercise Versus High-Intensity Interval Training on V̇O2max and Blood Pressure

**DOI:** 10.7759/cureus.30322

**Published:** 2022-10-15

**Authors:** Jean Tamayo Acosta, Ariel E Sosa Gomez, Steven Samuel, Stephanie Pelenyi, Rachel E Acosta, Marjorie Acosta

**Affiliations:** 1 Medicine, University of Medicine and Health Sciences, Bayamón, PRI; 2 Medical Student, Saint James School of Medicine, Miami, USA; 3 Medical Student, Avalon School of Medicine, Willemstad, CUW; 4 Research, Saint James School of Medicine, Miami, USA; 5 Psychiatry, Hospital Pavia Hato Rey, Bayamón, PRI

**Keywords:** maximun oxygen uptake, v̇o2max, exercise training, peak power output, aerobic exercise, heart rate peak, heart rate, diastolic blood pressure, systolic blood pressure, high intensity interval training

## Abstract

Today, more than 20% of the world's population suffers from hypertension, a major risk factor for heart disease. Therefore, lifestyle modifications such as dietary change, smoking cessation, and exercise are often prescribed to hypertensive patients as a first-line treatment. This study aims to examine and compare the effects of different exercise regimens on the cardiovascular system, particularly that of high-intensity interval training (HIIT) and lower-to-moderate-intensity aerobic exercise (aka aerobic exercise).

After researching various databases and extracting 4,724 studies, 196 were viable within the exclusion criteria related to exercise's effects on blood pressure and maximal oxygen uptake (V̇O_2_max). Of these, 36 studies were selected as viable, and their data is herein outlined. In addition, the results provided by these studies were summarized, respectively, and the raw data were analyzed using a two-tailed unpaired t-test. Through this study, we aim to clarify whether HIIT or lower-to-moderate-intensity aerobic exercise differ in their effects on improving cardiovascular health.

It was observed that HIIT was non-inferior to lower-to-moderate-intensity aerobic exercise in the reduction of ambulatory blood pressure of hypertensive or normotensive individuals. However, HIIT was more effective at increasing cardiorespiratory fitness by means of V̇O_2_max than aerobic exercise was. Considering the significant time-to-completion difference between both exercise modalities, it is remarkable that HIIT has the same benefits as lower-to-moderate-intensity aerobic exercise on blood pressure and higher efficiency in increasing V̇O_2_max.

## Introduction and background

The importance of exercise and its impact on human health has been extensively researched and well-documented [1]. The American College of Sports Medicine's (ACSM) position on exercise and hypertension (HTN) recommends dynamic aerobic endurance training for at least 30 minutes daily, preferably supplemented with dynamic resistance exercise [2]. Nonetheless, the effects of exercise training may vary with different exercise modalities (e.g., endurance training or resistance exercise) and dose parameters, specifically program length, session duration, frequency, and workload or intensity. As such, the optimal exercise training prescription remains unclear [3]. As new trials arise and our databases expand, it is vital to challenge our current knowledge and answer more elaborate questions. In a global community where cardiovascular disease is an increased risk and a major cause of death, researching and comparing the benefits of different exercise regimens on the cardiovascular system is beneficial.

The aim of this investigation was to compare the effects of high-intensity interval training (HIIT) versus low-to-moderate intensity continuous training on ambulatory blood pressure through a systematic review and analysis of both randomized controlled trials and other systematic review papers. HIIT generally refers to repeated sessions of relatively brief intermittent exercise, often performed with an "all-out" effort or at an intensity close to that which elicits V̇O_2_max (i.e., ≥90% of V̇O_2_max) [2]. V̇O_2_max is the highest value of oxygen consumption attained on a particular graded exercise test [2]. This is most commonly an incremental or other high-intensity test designed to bring the subject to the limit of tolerance and, as such, an appropriate measure of exercise intensity [4]. This paper will summarize the mean findings and effects of the respective exercises on various measures.

To understand the effects of HIIT on the cardiovascular system, measures of cardiovascular functioning, such as systolic blood pressure (SBP) and diastolic blood pressure (DBP), were observed and analyzed before and after the exercise regimens. SBP is a measure of the pressure in blood vessels when the heart contracts, while DBP relates to the pressure in the arteries when the heart relaxes between beats [5]. This investigation aims to determine the magnitude of change in SBP and DBP in subclinical and hypertensive populations after one session of exercise or after several weekly sessions. The null hypothesis states that there is no significant difference between HIIT and low-to-moderate intensity continuous training with respect to the effects on the cardiovascular system. Further, the study aims to examine whether the magnitude of change in SBP and DBP was related to exercise program characteristics, such as the number of sessions, duration, intensity, or exercise mode. Additionally, parameters, such as the effects of exercise on V̇O_2_max and angiogenesis, were also noted when available, under the premise that the body works harder after a bout of exercise to restore homeostasis [6]. Doing so increases V̇O_2_max over time and promotes the release of vascular endothelial growth factors from skeletal muscle, promoting angiogenesis.

## Review

Materials and methods

Data Sources

A total of 4,724 studies were identified through our database search. These were extracted from EBSCO, Pubmed/Medline, Cochrane, EMBASE: Excerpta Medica Database, and DARE electronic databases. After removing duplicates and two initial screening phases (title/abstract and full text), 4,431 studies were withheld based on the exclusion criteria. Of the remaining 293 articles, 92 viable articles published between the years 1990 and 2022 were identified. The others were excluded based on statistically insignificant information and/or failure to meet established parameters. Of these 92, a total of 56 articles were excluded as they were not relevant to the scope of this review. A total of 36 articles were then analyzed for the means of this paper.

Key search terms included exercise or aerobic exercise or high-intensity interval training and HTN or blood pressure or V̇O_2_max or moderate-intensity continuous training, or low-to-moderate intensity continuous training.

**Figure 1:**
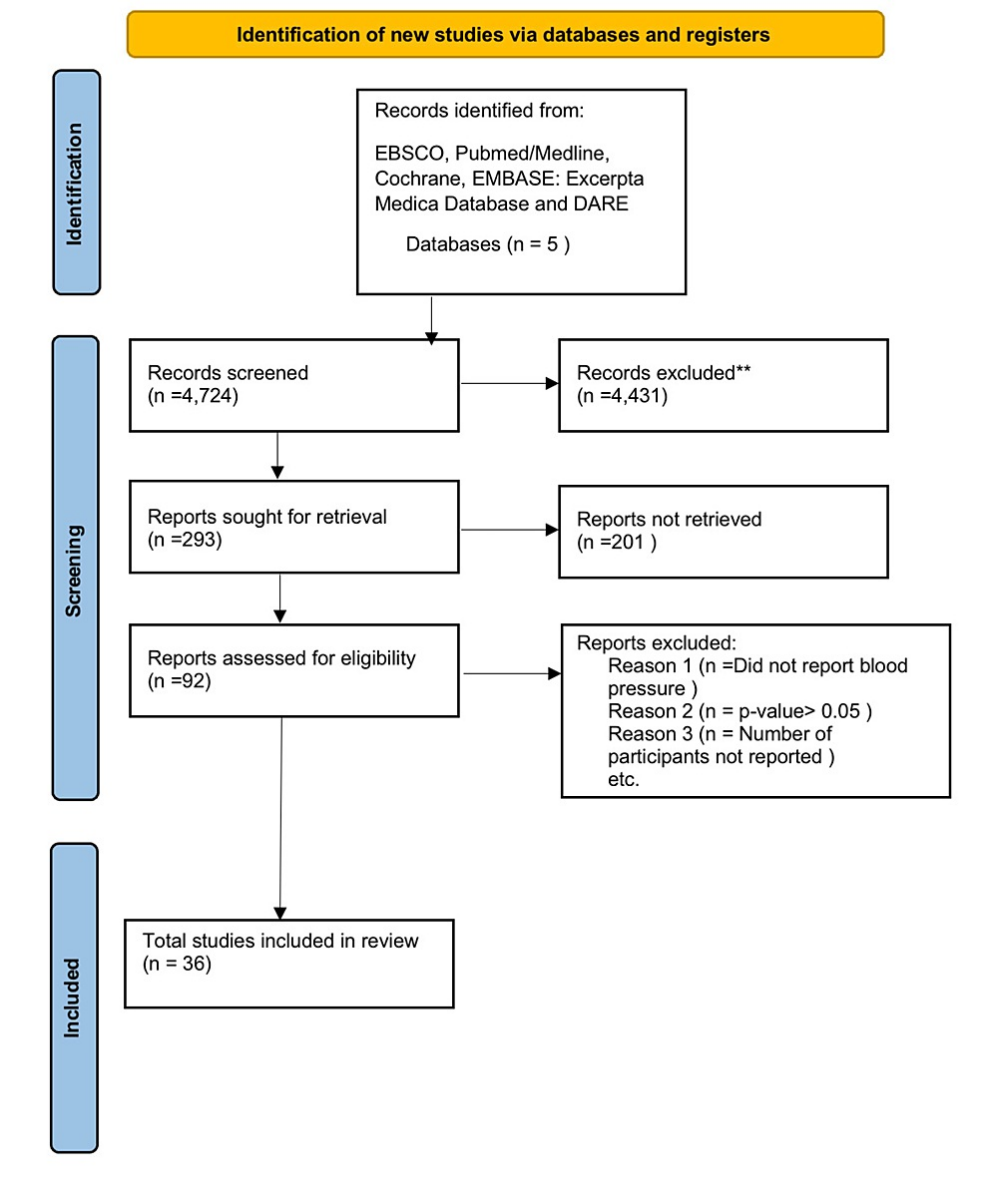
PRISMA diagram detailing study selection process.

The screening process is summarized in Figure 1.

Inclusion criteria

Studies were included in this research upon meeting the following criteria: the study must be written in English and must discuss the effect of low-to-moderate intensity continuous training or HIIT on blood pressure or V̇O_2_max on human adults (>18 years old). The study must be experimental, or a systematic review or meta-analysis of experimental studies on the effects of exercise on blood pressure or V̇O_2_max, or include measurements of the mentioned parameters. The study must include normotensive and/or hypertensive patients. The study must have been published after 1990. Studies excluded from this research were those that did not clearly state the number of participants; studies that did not include blood pressure or V̇O_2_max parameters; studies with diabetic patients or hypertensive patients with comorbid diseases; and studies were also excluded if they were reports, letters, or comments.

Data extraction

The authors gathered to formulate a research question and drafted a research proposal that included a protocol to be followed throughout the review. It included the databases, types of data, and parameters to be used prior to collecting data. The search terms set in the inclusion/exclusion criteria above were followed upon extraction and review of the studies. Each author was responsible for searching at least one of the aforementioned databases. After the extraction, each author was responsible for ensuring that all citations for their respective studies were included in the appropriate format, there were no duplicates, and all studies were accessible through the respective databases. It was ensured that all data acquired were from credible academic databases and that all material had been cited lawfully. Having multiple reviewers ensured that information was reported accurately. The selected participants and values were obtained from per-protocol numbers. Individuals that failed to complete their respective regimens were excluded from calculations. Data from papers that failed to provide statistically significant results or were simply not reported were excluded from calculations. 

The authors extracted and summarized all related data from the selected studies and detailed it in Tables below.

Statistical analysis

The statistical analysis was conducted using a two-tailed unpaired t-test for computing variances and p-values, which were set for a statistical significance <0.05. Mean values were calculated using the data for each of the groups, HIIT vs. low-to-moderate intensity continuous training for each parameter V̇O_2_max, SBP, and DBP.

Results

All studies included in this analysis employed a HIIT intervention or a low-to-moderate intensity continuous training regimen. The number of study participants in HIIT groups ranged from 6 to 183. Most participants were young (18-45 years old) men and women, both normotensive and hypertensive. The HIIT protocols ranged from a single acute session to longer-term multiple sessions (four HIIT sessions, lasting 4 minutes per session, three times per week for 12 weeks). Low-to-moderate intensity continuous training modalities (at ~65% V̇O_2_max) included treadmill running, swimming, and cycling. HIIT exercise modalities (at >90% V̇O_2_max) included bouts of exercise at high intensity interspersed with low-intensity activity.

Effect of HIIT on the cardiovascular system

Table 1 summarizes the effects of HIIT on the cardiovascular system in terms of V̇O_2_max and blood pressure. A total of 17 papers described the effect of HIIT on V̇O_2_max. 

**Table 1:** The summarized results of studies that entailed measuring the defined parameters regarding the effect of high-intensity interval training on the cardiovascular system. HIIT: High-intensity interval training; V̇O2max: Maximum rate of oxygen uptake; SBP: Systolic blood pressure; DBP: Diastolic blood pressure; HR: Heart rate; HRpeak: Heart rate peak; HRmax: Maximum heart rate; VT2: Second ventilatory threshold; vVT2: Velocity associated with VT2; vV̇O2max: Velocity at maximal oxygen uptake; PPO: Peak power output. Please note that the sections with no values have been marked with a (-) to indicate that the featured study did not provide a value for the respective parameter. In addition, values that were not statistically significant (p-value >0.05) were also excluded and marked with a (-). The number of sessions with ranging values describes meta-analyses with varying modalities included in their respective studies. * Studies that describe hypertensive patients have been marked with an asterisk.

Study	Authors	Participant number	V̇O_2_max Increase	Effect on SBP	Effect on DBP	Exercise Method	Year	Exercise Sessions
Effects of HIIT compared to moderate-intensity continuous training on maximal oxygen consumption and blood pressure in healthy men: A randomized controlled trial	Arboleda-Serna VH et al. [7]	22	9.40%	-	-	15 bouts of 30 seconds (90-95%, maximal heart rate)	2019	24
HIIT versus traditional exercise interventions for promoting health	Nybo L et al. [8]	35	12.00%	-8	-2	The high-intensity training consisted of a brief 5-min warm-up with light jogging followed by five intervals of 2 min of near-maximal running (95% HR max)	2010	36
HIIT improves health and physical function in middle-aged adults	Adamson S et al. [9]	8	8.00%	-4	-2	16 sessions of HIIT were spread over an eight week period, with one or two days of rest between each sprint.	2014	1
Effects of HIIT versus moderate-intensity continuous training on blood pressure in adults with pre- to established hypertension: a systematic review and meta-analysis of randomized trials	Costa EC et al. [10]	143	13.30%	-6.3	-3.8	Repeated high-intensity interval bouts between 80% and 100% of HRpeak interspersed with recovery periods or light exercise	2018	36-48
Is oxygen uptake measurement enough to estimate energy expenditure during high-intensity intermittent exercise? Quantification of anaerobic contribution by different methods	Panissa VL et al. [11]	13	2.90%	-	-	10 1-min efforts completed at vVO˙ 2 max separated by 1-min of passive recovery on the treadmill.	2018	6
HIIT improves resting blood pressure, metabolic (MET) capacity and heart rate reserve without compromising cardiac function in sedentary aging men	Grace F et al. [12]	39	10.00%	-7.7	-4.6	6 x 30 cycle sprints @ 40% PPO; interspersed by 3 min recovery intervals X once every 5 days	2017	9
Exercise training for blood pressure: a systematic review and meta-analysis	Cornelissen VA et al. [13]	77	-	-3.18	-3.07	Various HIIT modalities	2013	36-364
Effectiveness of high-intensity interval training versus moderate-intensity continuous training in hypertensive patients: a systematic review and meta-analysis	Leal JM et al. [14]	183	10.90%	-5.64	-4.8	Various HIIT modalities	2020	36-48
Effects of a high-intensity interval training program versus a moderate-intensity continuous training program on maximal oxygen uptake and blood pressure in healthy adults: study protocol for a randomized controlled trial	Arboleda Serna VH et al. [15]	10	-	-3.76	-2.94	20 min of treadmill running with a 1:4 min work to rest ratio, an upper HR target at 80–85% of HR-reserve, and a lower HR target at 40–60% of HR-reserve	2022	12
Low- and high-volume of intensive endurance training significantly improves maximal oxygen uptake after 10-weeks of training in healthy men	Tjønna AE et al. [16]	24	11.50%	-6.2	-7.7	10-min warm-up followed by four, 4-min intervals at ∼90% of HRmax interspersed by 3 min of active recovery at ∼70% of HRmax,	2008	36-48
Short duration high-intensity interval training improves aerobic conditioning of female college soccer players	Rowan AE et al. [17]	6	4.45%	-	-	Five repetitions of 30-sec maximum effort sprints with 4.5 min active recovery (jogging) for a total of 25 minutes	2012	10
Whole-body high-intensity interval training induce similar cardiorespiratory adaptations compared with traditional high-intensity interval training and moderate-intensity continuous training in healthy men	Schaun GZ et al. [18]	15	9.80%	-	-	4-minute warm-up at 90–95% vVT2. Followed by 20-second bouts at 130% vV̇O_2_max interspersed by 10 seconds of passive recovery	2018	78
Twelve weeks of sprint interval training improves indices of cardiometabolic health similar to traditional endurance training despite a five‐fold lower exercise volume and time commitment	Gillen JB et al. [19]	27	19.00%	-	-	3x20-second ‘all-out’ cycle sprints (~500W) interspersed with 2 minutes of cycling at 50W	2016	31
Effect of high-intensity interval training on body composition, cardiorespiratory fitness, blood pressure, and substrate utilization during exercise among prehypertensive and hypertensive patients with excessive adiposity	Delgado-Floody P et al. [20]	23	-	-6.98	-	1 min of maximum intensity exercise using a magnetic resistance static bicycle followed by 2 min of passive recovery over the bicycle; repeated 10 times	2020	36
Aerobic interval training reduces blood pressure and improves myocardial function in hypertensive patients	Molmen-Hansen HE et al. [21]	25	15%	-12	-8	10 minutes warm up at ∼60% of HRmax, followed by 4 × 4 minute intervals at 90–95% of HRmax, with 3 minutes active pause between the exercise bouts at 60–70% HRmax.	2012	36
Aerobic interval training and continuous training equally improve aerobic exercise capacity in patients with coronary artery disease: the SAINTEX-CAD study	Conraads VM et al. [22]	85	22.70%	-	-1.1	4 sets × 4 min 90%-95% HRpeak, 3 min active recovery	2015	36
Continuous exercise but not high intensity interval training improves fat distribution in overweight adults	Keating SE et al. [23]	11	16.80%	-5.6	-2.9	60 seconds cycle:120 seconds rest	2014	36
High intensity interval training in a realworld setting: a randomized controlled feasibility study in overweight inactive adults, measuring change in maximal oxygen uptake	Lunt H et al. [24]	18	4.50%	-4.7	-4.4	Various HIIT Modalities	2014	36
Twelve weeks of low volume sprint interval training improves cardio-metabolic health outcomes in overweight females	Sun S et al. [25]	14	25%	-	-	9 × 4-min cycling at 90% V̇O_2_peak + 3-min rest	2018	36
Impact of low-volume, high-intensity interval training on maximal aerobic capacity, health-related quality of life and motivation to exercise in ageing men	Knowles et al [26]	39	10.50%	-	-	[6 x 30-s cycle sprints interspersed by 3-min recovery intervals	2015	9
Total Participants		818						
Total Papers		20						

Table 2 outlines the average results for the V̇O_2_max after the HIIT exercise. After working out for 1-10 sessions, there was a 7.17% average increase from the established V̇O_2_max baseline. The average result for the V̇O_2_max after working out for 11-36 sessions was a 16% increase, and the average result for the V̇O_2_max after working out for 37+ sessions was an 11.38% increase. Papers in this session that provided no V̇O_2_max values were excluded from the calculations. A total of 13 studies described the effects of HIIT on blood pressure. Of these, the mean for the SBP after working out with the HIIT regimen for 1-10 sessions showed a 5.85 mmHg decrease in blood pressure. The mean for the SBP after working out for 11-36 sessions was a 6.84 mmHg decrease. The mean for the SBP after working out for 37+ sessions was a 5.33 mmHg decrease. The mean for the DBP for 1-10 sessions was a 3.30 mmHg decrease. The mean for the DBP (11-36 sessions) was a 3.56 mmHg decrease, and the mean for the DBP for 37+ sessions was a 4.82 mmHg decrease. Values that have been replaced by a (-) in this session were excluded, given that no measurements were provided to meet the necessary parameters or no statistical significance was found by the researchers of their respective papers.

**Table 2:** Describes the average effect of HIIT modalities on the maximum rate of oxygen uptake, systolic blood pressure, and diastolic blood pressure, as related to the number of sessions performed. HIIT: High-intensity interval training; V̇O_2_max: Maximum rate of oxygen uptake; SBP: Systolic blood pressure; DBP: Diastolic blood pressure. *Papers used for calculations given that the respective parameters were met and statistical significance was provided in the results.

HIIT Sessions	1-10	11-36	37+	1-10	11-36	37+	1-10	11-36	37+
*Number of papers within parameters	5	8	4	2	6	4	2	6	4
	V̇O_2_max	V̇O_2_max	V̇O_2_max	SBP	SBP	SBP	DBP	DBP	DBP
Average	+7.17%	+16.00%	+11.38%	-5.85	-6.84	-5.33	-3.30	-3.56	-4.82

It was observed that HIIT increases V̇O_2_max by an average of 11.52% with a p-value of 0.0107. However, the extent of the increase varies depending on the number of sessions performed. We observed that while 11-36 sessions over the extent of approximately 12 weeks, this is to say, a maximum of three sessions a week yielded a higher increase in V̇O_2_max than performing more than three sessions a week or performing HIIT consistently over a prolonged period of time. The same distribution in values was observed in the mean reduction of SBP. Performing 11-36 sessions over a 12-week period appeared to have a greater effect on SBP than performing more sessions, or less than these, for that matter. The overall reduction of SBP by using HIIT in both hypertensive and normotensive patients was -6.00 on average, with a p-value of 0.0053; the authors noted that the greatest reductions were observed in hypertensive patients as described by the aforementioned studies.

Effect of low-to-moderate intensity continuous training on the cardiovascular system

Table 3 summarizes the effects of low-to-moderate intensity continuous training on the cardiovascular system in terms of V̇O_2_max and blood pressure. A total of eight papers described the effect of low-to-moderate intensity continuous training on V̇O_2_max.

**Table 3:** Studies featuring the effect of low-to-moderate intensity continuous training on the cardiovascular system. HIIT: High-intensity interval training; V̇O2max: Maximum rate of oxygen uptake; SBP: Systolic blood pressure; DBP: Diastolic blood pressure; HR: Heart rate; HRpeak: Heart rate peak; HRmax: Maximum heart rate; VT2: Second ventilatory threshold; vVT2: Velocity associated with VT2; vV̇O2max: Velocity at maximal oxygen uptake; PPO: Peak power output. Please note that the sections with no values have been marked with a (-) to indicate that the featured study did not provide a value for the respective parameter. In addition, values that were not statistically significant (p-value >0.05) were also excluded and marked with a (-). The number of sessions with ranging values describes meta-analyses with varying modalities included in their respective studies. * Studies that describe hypertensive patients have been marked with an asterisk.

Study	Authors	Participants number	V̇O_2_max Increase	Effect on SBP	Effect on DBP	Exercise Method	Year	Exercise Sessions
Effects of HIIT compared to moderate-intensity continuous training on maximal oxygen consumption and blood pressure in healthy men: A randomized controlled trial	Arboleda-Serna VH et al. [7]	22	6%	-0.7	-	Completed 40 minutes of continuous exercise (65-75% HRmax)	2019	24
HIIT versus traditional exercise interventions for promoting health	Nybo L et al. [8]	36	6.8%	-8	-5	60 minutes of continuous running at 80% of individual HR max	2010	36
Effects of high-intensity interval training versus moderate-intensity continuous training on blood pressure in adults with pre- to established hypertension: a systematic review and meta-analysis of randomized trials	Costa EC et.al [10]	127	11.20%	-5.8	-3.5	Treadmill exercise for 40 min	2018	36-48
Exercise training for blood pressure: a systematic review and meta-analysis	Cornelissen VA and Smart NA [13]	37	-	-4.58	-2.66	Various low-to-moderate intensity continuous training modalities	2013	36-364
Effectiveness of high-intensity interval training versus moderate-intensity continuous training in hypertensive patients: a systematic review and meta-analysis	Leal JM et al. [14]	185	3.15%	-3.7	-2.41	Various low-to-moderate intensity continuous training modalities	2020	36-48
Effects of a high-intensity interval training program versus a moderate-intensity continuous training program on maximal oxygen uptake and blood pressure in healthy adults: study protocol for a randomized controlled trial	Arboleda Serna VH et.al [15]	10	-	-1.67	-1.5	20 min of treadmill at a lower HR target at 40–60% of HR-reserve	2022	12
Short duration high-intensity interval training improves aerobic conditioning of female college soccer players	Rowan AE et al. [17]	5	3.89%	-	-	40- min run at 80% of V̇O_2_max	2012	10
Whole-body high-intensity interval training induce similar cardiorespiratory adaptations compared with traditional high-intensity interval training and moderate-intensity continuous training in healthy men	Schaun GZ et al. [18]	14	15.40%	-	-	30 minutes on a motorized treadmill at an intensity corresponding to 90–95% of the HR associated with VT2	2018	48
Twelve weeks of sprint interval training improves indices of cardiometabolic health similar to traditional endurance training despite a five‐fold lower exercise volume and time commitment	Gillen JB et al. [19]	27	19%	-	-	45 minutes of continuous cycling at ~70% HRmax	2016	32
Aerobic interval training reduces blood pressure and improves myocardial function in hypertensive patients	Molmen-Hansen HE et al. [21]	23	5%	-4.5	-3.5	Walking/running on treadmill at 70% of HRmax (corresponding to 60% of V̇O_2_max), for 47 minutes	2012	36
Aerobic interval training and continuous training equally improve aerobic exercise capacity in patients with coronary artery disease: the SAINTEX-CAD study	Conraads VM et al. [22]	89	20.30%	-6	-3.7	3 day/week 37 min at 70%- 75% %HRmax	2015	36
Continuous exercise but not high intensity interval training improves fat distribution in overweight adults	Keating SE et al. [23]	11	15.20%	-4.3	-3.5	45 minutes at an intensity of 65% of V̇O_2_peak	2014	36
High intensity interval training in a real world setting: a randomized controlled feasibility study in overweight inactive adults, measuring change in maximal oxygen uptake	Lunt H et al. [24]	14	4.80%	-5.2	-1.9	33 minute walking at 65-75% HRmax	2014	36
Twelve weeks of low volume sprint interval training improves cardio-metabolic health outcomes in overweight females	Sun S et al. [25]	14	25%	-	-	cycling at 60% V̇O_2_peak for ~ 61-min	2018	36
The magnitude and duration of ambulatory blood pressure reduction following acute exercise	Wallace JP et al. [27]	46	-	-6.8	-4.1	Treadmill 50min	1999	1
Acute effects of continuous and interval aerobic exercise on 24-h ambulatory blood pressure in long-term treated hypertensive patients	Ciolac EG et al. [28]	52	-	-2.6	-2.3	Cycling 40min	2009	1
After effects of exercise on regional and systemic hemodynamics in hypertension	Cléroux J et al. [29]	22	-	-11	-4	30 min cycling	1992	1
Effects of aerobic exercise on blood pressure: a meta analysis of randomized controlled trials	Whelton SP et al. [30]	2491	-	-3.84	-2.58	Aerobic exercise	2002	1
Blood pressure measurements in research: suitability of auscultatory, beat-to-beat, and ambulatory blood pressure measurements	Carlson DJ et al. [31]	50	-	-2.9	-2.1	40 min cycling	2008	1
Controlled trial of aerobic exercise in hypertension	Martin JE et al. [32]	10	-	-6.4	-9.6	lLow-to-moderate intensity continuous training for 30 min	1990	4
Effects of weight on blood pressure at rest and during exercise	Schoenenberger AW et al. [33]	17	-	-17.5	-8.1	25 min cycling	2013	1
Isolated and combined effects of aerobic and strength exercise on post-exercise blood pressure and cardiac vagal reactivation in normotensive men	Ruiz RJ et al. [34]	11	-	-4.8	-2.8	40 minutes of cycling between 60 and 70% of HR Reserve on a cycle ergometer	2011	36
Controlled trial of aerobic exercise in hypertension	Martin JE et al. [32]	12	-	-6	-9	Aerobic exercise, cycling	1991	3
A comparison of the immediate effects of resistance, aerobic, and concurrent exercise on postexercise hypotension	Keese F et al. [35]	21	-	-6.3	-1.8	50 mins of aerobic cycling	2013	4
Aerobic exercise reduces blood pressure in resistant hypertension	Dimeo F et al. [36]	50	-	-6	-3	Treadmill for 20-75 min	2012	12
The effectiveness of exercise training in lowering blood pressure: a meta-analysis of randomized controlled trials of 4 weeks or longer	Halbert JA et al. [37]	1533	--	-4.7	-3.1	Treadmill 40 min	1997	12
Ambulatory blood pressure after acute exercise in older men with essential hypertension	Taylor-Tolbert NS et al. [38]	11	--	-7.4	-3.6	Treadmill for 45 min	2000	30
Effects of high-intensity circuit training, low-intensity circuit training and endurance training on blood pressure and lipoproteins in middle-aged overweight men	Paoli A et al. [39]	58	--	-4	-3	Cycling for 50 min	2013	36
Acute and chronic effects of aerobic and resistance exercise on ambulatory blood pressure	Cardoso CG Jr et al. [40]	341	-	-3.3	-3.5	Treadmill 50min	2010	36
Separate effects of intensity and amount of exercise on interindividual cardiorespiratory fitness response	Ross R et al. [41]	121	3.20%	-		Treadmill 40 min walk	2015	120
Total Participants		5460						
Total Papers		30						

Table 4 summarizes the results of the obtained means for the effects of low-to-moderate intensity continuous training on the cardiovascular system regarding blood pressure and V̇O_2_max. After working out for 1-10 sessions, there was a 3.9% average increase from the established V̇O_2_max baseline. The average result for the V̇O_2_max after working out for 11-36 sessions was a 14% increase, and the average result for the V̇O_2_max after working out for 37+ sessions was an 8.2% increase. Papers in this session that provided no V̇O_2_max values were excluded from the calculations. A total of 25 studies described the effects of low-to-moderate intensity continuous training on blood pressure. Of these, the mean for the SBP after working out with the low-to-moderate intensity continuous training regimen for 1-10 sessions showed a 7.0 mmHg decrease in blood pressure. The mean for the SBP after working out for 11-36 sessions was a 4.7 mmHg decrease. The mean for the SBP after working out for 37+ sessions was a 4.7 mmHg decrease. The mean for the DBP after working out for 1-10 sessions was a 4.8 mmHg decrease. The mean for the DBP (11-36 sessions) was a 3.2 mmHg decrease, and the mean for the DBP for 37+ sessions was a 2.8 mmHg decrease. Values that have been replaced by a (-) in this session were excluded given that no measurements were provided to meet the necessary parameters or no statistical significance was found by the researchers of their respective papers.

**Table 4:** Average effect of low-to-moderate intensity continuous training modalities on the maximum rate of oxygen uptake, systolic blood pressure, and diastolic blood pressure, as related to the number of sessions performed. HIIT,  High Intensity Interval Training; V̇O_2_max, maximum rate of oxygen uptake; SBP, systolic blood pressure; DBP, diastolic blood pressure. *Papers used for calculations given that the respective parameters were met and statistical significance was provided in the results.

Aerobic Sessions	1-10	11-36	37+	1-5	6-36	37+	1-5	6-36	37+
*Number of papers within parameters	1	8	4	9	13	3	9	12	3
	V̇O_2_max	V̇O_2_max	V̇O_2_max	SBP	SBP	SBP	DBP	DBP	DBP
Average	3.9%	14%	8.2%	-7.0	-4.7	-4.7	-4.8	-3.2	-2.8

It was observed that low-to-moderate intensity continuous training increases V̇O_2_max by an average of 8.73%, with a p-value of 0.041. The extent of the increase, similar to what was observed in HIIT, varied depending on the number of sessions performed. We observed that while 11-36 sessions over the extent of approximately 12 weeks, this is to say, a maximum of 3 sessions a week, yielded a higher increase in V̇O_2_max than performing more than three sessions a week or performing low-to-moderate intensity continuous training consistently over a prolonged time. This value distribution was not observed in the mean reduction of SBP. Performing 11-36 sessions over a 12-week period appeared to have the same effect as performing 37+ sessions, both effects being reportedly lower than the initial drop in SBP observed with 1-10 sessions. The overall reduction of SBP by using HIIT in both hypertensive and normotensive patients was -5.47 on average with a p-value of 0.002.

Discussion

With the widespread increase in morbidity and mortality in hypertensive patients, more importance has been placed on preventing the disease and finding better ways to manage it [1]. One of the most commonly given adjunct treatments is a lifestyle change, including modifications to diet, alcohol intake, smoking exposure, and exercise regimen [1]. In addition, numerous studies have shown evidence that supports the use of increased physical activity [1] to decrease and control the development of HTN [42]. To understand multifactorial pathways for how exercise reduces the risk of and protects against cardiovascular disease, parameters such as systemic blood pressure and V̇O_2_max were utilized. These were used to compare the efficiency of HIIT (at ~90% V̇O_2_max) versus low-to-moderate intensity continuous training (at ~66% V̇O_2_max).

HIIT versus low-to-moderate intensity continuous training on VO_2_max

It was noted in this study that the mean values for the effect of HIIT on V̇O_2_max gradually increased per the number of sessions completed. However, as described herein, it was also noted that while there was a benefit to increased sessions, this benefit was to a lesser extent than that of performing approximately three exercise sessions a week. There was an overall increase in V̇O2max for HIIT and aerobic exercise. However, HIIT proved to have a more significant effect than traditional aerobic training on V̇O2max (p<0.02). These results are consistent with recent studies, which have found that "high-intensity aerobic interval training resulted in significantly increased V̇O2max compared with long slow distance and lactate-threshold training intensities (P <0.01)" [43]. A two-tailed unpaired t-test was performed to compare the average effect of HIIT on V̇O_2_max versus the average effect of aerobic training on V̇O_2_max, resulting in P<0.0204. It was observed that HIIT was superior to low-to-moderate intensity continuous training in increasing V̇O_2_max at all session intervals. However, no statistical significance was found regarding the superiority of either HIIT or low-to-moderate intensity continuous training and their respective effects on reducing SBP or DBP. This is to say that in the matter of blood pressure reduction, HIIT was found to be non-inferior to low-to-moderate intensity continuous training. Of note, the average time for the performance of HIIT modalities was approximately 10-15 min for the majority of papers included in this study, whereas the time taken to perform low-to-moderate intensity continuous training sessions ranged in the 40-60 minutes mark. Figure 2 compares the increase in average V̇O_2_max based on the type of exercise, HIIT vs. aerobic exercise, and the number of sessions.

HIIT versus low-to-moderate intensity continuous training on blood pressure

The pooled data suggests to the authors that while both HIIT and low-to-moderate intensity continuous training decrease the blood pressure of both hypertensive and normotensive patients, there is no statistically significant difference as to the extent to which they do so. Meaning HIIT is non-inferior to low-to-moderate intensity continuous training in reducing blood pressure, thus rejecting the hypothesis that HIIT may have a higher impact on blood pressure than aerobic exercise. These findings are consistent with previous studies, which have found that, in contrast to cardiorespiratory fitness, exercise's acute and chronic effects on resting and ambulatory BP appear not to be influenced by exercise intensity [44,45]. In other words, while higher-intensity exercise regimens appear to have a significant effect on cardiorespiratory fitness, it has been observed that the effect on blood pressure reduction is not intensity-dependent. It is of note that studies have shown that the most common barrier to patient compliance to prescribed patient exercise is the lack of time [44]. The decreased time it takes to achieve HIIT versus low-to-moderate intensity continuous training does make the prospect of considering HIIT more attractive to individuals with a busier schedule.

When looking at mean values of the number of sessions performed in aerobic exercises, the mean values did not alter significantly with gradual session quantity increase; this is to say that patients did not experience any statistically significant decrease in blood pressure per session. The pooled data suggests to the authors that implementing either HIIT or low-to-moderate intensity continuous training regimens over long periods or at a higher frequency within a limited period does increase cardiorespiratory fitness in terms of V̇O_2_max, as well as decreasing blood pressure. However, these benefits do not appear to exhibit direct proportionality between the extent of the benefit and the number of workout sessions. Some studies [20] noted the difference in BP between HTN and non-HTN patients; the decrease in SBP was observed to be more significant if the patients were hypertensive before the exercise regime. Furthermore, there were studies in which a portion (~24%) of the subjects in the HIIT group became normo-tone (<130 mmHg systolic). In contrast, a smaller portion (4.34%) low-to-moderate intensity continuous training group and one person in the control group obtained normotensive values after the study period [21]. These findings are also consistent with other studies, which showed that more intense HIIT modalities yielded a higher reduction in SBP and DBP than lower-intensity HIIT modalities [24]. While there is no significant difference between HIIT and low-to-moderate intensity continuous training in blood pressure reduction, higher-intensity modalities appear to have a slightly greater effect on the blood pressure reduction of previously hypertensive patients.

HTN is a progressive disease capable of establishing many life-threatening complications, including, but not limited to, congestive heart failure, strokes, renal failure, or myocardial infarctions [46]. Some of the most common treatments are lifestyle changes, including modifications to diet, alcohol intake, smoking exposure, and exercise regimen, often combined with drug therapy [46]. Patient compliance is a major issue that arises from prescribing exercise as a lifestyle modification [47]. The most commonly used low-to-moderate intensity continuous training regimens involve moderate activity and require a sustained duration of at least thirty minutes [3]. This review presents statistical evidence rejecting the null hypothesis that "there is no significant difference between exercise modalities of short duration such as HIIT and longer duration such as traditional aerobic exercise, concerning the effects on the cardiovascular system." HIIT requires significantly less time to perform and may result in higher patient compliance. The differences in studies between HIIT compared with low-to-moderate intensity continuous training at lower intensities were analyzed in this study.

Limitations

The authors of this systematic review are aware that there is a limited volume of data and participants in studies concerning the effects of exercise on HTN. An unconsidered issue was the multifactorial pathways of how exercise affects blood pressure, as these are not yet fully understood and may play an important role in the disease progression. The lack of differentiation between younger and older adults and the lack of details portraying the differences between genders is also considered a limitation of this study. Some research studies included acute exercise impact performance with specific V̇O_2_max on the cardiovascular response for a specific recovery time. However, they failed to mention any pre-existing health conditions that might have impacted the final results. Additionally, there was a failure to assess the effect of the intervention of regular physical activity, eating habits, and continued compliance with HIIT and low-to-moderate intensity continuous training protocols daily in all the included studies. In most of the HIIT studies, there was no documentation of the time between the last training session and the blood pressure recording. There was also a lack of reporting regarding adherence to the designed regiment or frequent supervision of adverse events. Differences in the natural aging process, such as hormonal changes, can have undocumented effects on bodily processes, and as such, differences in data may be misleading. The authors concluded that some of the limitations contained enough data values that were a suitable match for the assigned parameters. In research, it can prove difficult to find the necessary data for the guidelines of a specific protocol. This may have significantly impacted the results of the mean value comparison by not having sufficient data to perform a complete comparison among all the studies included in this review. The authors, however, strived to maintain the data as accurate as possible through a rigorous methodology and multi-layered verification of all calculations to avoid skewing the results.

## Conclusions

The described findings provide compelling reasons to recommend HIIT as an alternative to patients being prescribed exercise as a treatment. This would better fit scheduling and time constraints while significantly reducing the risk of developing hypertension and, in some cases eliminating high blood pressure. However, future research must be done regarding how exercise affects the cardiovascular system, as well as research describing the effect of various exercise regimes on preventing other comorbidities. With this article, we intend to confirm that there are viable, time-efficient alternatives to the traditional low-to-moderate intensity continuous training regime, which is so often recommended as part of lifestyle modifications for hypertensive and pre-hypertensive patients.
